# Pericardiocentesis, a Stress-Relieving Procedure Causing a Paradoxical Effect: A Case Report on Stress-Induced Takotsubo Cardiomyopathy

**DOI:** 10.7759/cureus.77773

**Published:** 2025-01-21

**Authors:** Rayan M Ghazal, Khalid O Almutlaq, Owais Mohammed Rahim, Ramy A Elesely

**Affiliations:** 1 Medicine, King Khalid University Hospital, Riyadh, SAU; 2 Cardiology, King Khalid University Hospital, Riyadh, SAU

**Keywords:** left ventricular apical ballooning, left ventricular systolic dysfunction, pericardial effusion, pericardiocentesis, stress-induced cardiomyopathy, takotsubo cardiomyopathy, tamponade

## Abstract

Takotsubo cardiomyopathy (TC), also known as stress cardiomyopathy, is a transient cardiac condition characterized by reversible left ventricular dysfunction, typically triggered by physical or emotional stress. While commonly associated with non-procedural stressors, TC has also been observed following medical or surgical interventions. This case report highlights TC as a rare but significant complication to a relatively safe and often life-saving procedure such as pericardiocentesis, underscoring the importance of timely recognition and management. Increased awareness of its potential triggers can prompt further research to ultimately enhance understanding of its pathophysiology, especially when occurring due to procedural stressors, to dedicate management plans directed toward the pathophysiology resulting in improved patient outcomes.

## Introduction

Takotsubo cardiomyopathy (TC), also known as stress-induced cardiomyopathy, is a temporary and reversible condition characterized by left ventricular systolic dysfunction, first described in 1990 [1]. It typically mimics myocardial infarction in the sense that both present with cardiac chest pain and have ST elevation or other ECG changes, but TC occurs in the absence of coronary artery disease [1]. TC is often precipitated by physical or emotional stressors [1,2], though it has also been linked to medical or surgical interventions.

The diagnostic criteria for TC, as outlined by the Heart Failure Association (HFA) in 2015, are as follows: 1.﻿﻿﻿ Transient regional wall motion abnormalities of left ventricular (LV) or right ventricular (RV) myocardium, which are frequently, but not always, preceded by a stressful trigger (emotional or physical); 2. ﻿﻿﻿The regional wall motion abnormalities usually extend beyond a single epicardial vascular distribution, and often result in circumferential dysfunction of the ventricular segments involved; 3. ﻿﻿﻿The absence of culprit atherosclerotic coronary artery disease including acute plaque rupture, thrombus formation, and coronary dissection or other pathological conditions to explain the pattern of temporary LV dysfunction observed (e.g. hypertrophic cardiomyopathy, viral myocarditis); 4. ﻿﻿﻿New and reversible electrocardiography (ECG) abnormalities (ST-segment elevation, ST depression, left bundle branch block (LBBB), T-wave inversion, and/or QTc prolongation) during the acute phase (three months); 5. ﻿﻿﻿Significantly elevated serum natriuretic peptide (brain natriuretic peptide (BNP) or N-terminal pro-B-type natriuretic peptide (NT-proBNP)) during the acute phase; 6. ﻿﻿﻿Positive but relatively small elevation in cardiac troponin measured with a conventional assay (i.e. disparity between the troponin level and the amount of dysfunctional myocardium present); ﻿﻿﻿7. Recovery of ventricular systolic function on cardiac imaging at follow-up (three to six months) [1].

## Case presentation

Patient history

The patient was a 54-year-old woman with a history of hypertension and end-stage kidney disease (ESKD), managed with peritoneal dialysis for several years. She had no prior cardiac history, including coronary artery disease, heart failure, or arrhythmias. The patient presented to the emergency department with acute-onset shortness of breath that had progressively worsened over the past day. She denied chest pain, cough, palpitations, fever, loss of consciousness, or a history of similar episodes. She also denied contact with sick patients.

Her home medications included: Nifedipine extended-release, 120 mg OD; Sevelamer carbonate, 1600 mg TID with meals (WM); Lasix 120 mg BID; Valsartan 160 mg OD; Calcium carbonate, 600 mg oral TID; Carvedilol, 6.25 mg oral, q12hr; and Darbepoetin alfa 40 mcg subcutaneously, qWeekly.

Physical examination

On arrival, the patient was hemodynamically stable, with a heart rate of 73, blood pressure of 119/88 mmHg, and O_2_ saturation of 99% on room air. She was afebrile, conscious, alert, and oriented. Physical examination revealed elevated jugular venous pressure and muffled heart sounds. She had equal bilateral air entry, with no pulmonary rales, added sounds, or other findings suggestive of volume overload or acute pulmonary congestion. Her abdomen was soft and lax, with no tenderness. There were no signs of peripheral edema. The peritoneal dialysis site was examined, and there was no discoloration, discharge, or pain.

Initial investigations

**Figure 1:**
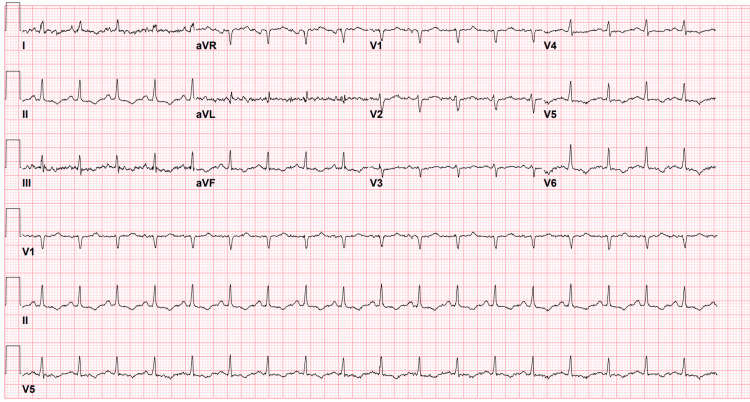
ECG on first presentation Sinus rhythm and low voltage in the precordial leads with poor R-wave progression, along with T-wave inversion in inferior leads only, but no evidence of ST-segment changes ECG: electrocardiogram

Bedside echocardiography revealed a large pericardial effusion with tamponade physiology (including systolic atrial collapse and diastolic right ventricular collapse, along with a plethoric inferior vena cava). Electrocardiography revealed sinus rhythm and low voltage in the precordial leads with poor R-wave progression, along with T-wave inversion in inferior leads only, but no evidence of ST-segment changes (Figure 1). Electrolytes and hemoglobin levels were within normal limits (Table 1), consistent with the patient’s baseline laboratory values in the context of ESKD. Laboratory tests demonstrated an elevated troponin level of 133 and a BNP level of 6000 pg/ml (Table 2).

**Table 1:** Summary of laboratory results ER: emergency room

ER Labs		Normal values	ER Labs		Normal values
WBC	9.060	4.5-11	Cr	1355	53-92
Hgb	10.1	12-16	BUN	22.8	2.5-10
Hct	31.2	36-46	Glucose	7.03	<5.6
PLT	412	150-400	Phos	3.11	0.97-1.45
Na	134	135-145	Ca	2.01	2.25-2.5
K	4.35	3.5-5.2	ALT	41.4	4-36
CO2	18	22-29	AST	25.7	8-33
Cl	91.4	96-106			

**Table 2:** Summary of laboratory results ER: emergency room; S/P: status post

	Troponin-T (ng/L)	BNP (pg/ml)
ER (pre-pericardiocentesis)	133.7	6022
Post-pericardiocentesis	246	6829
Day 1 S/P	1892	
Day 2 S/P	1407	
Day 3 S/P	1649	

Intervention

Given the echocardiographic findings of impending tamponade, percutaneous pericardiocentesis was performed under a sterile technique with echocardiographic guidance via the subxiphoid approach. Initially, 1250 mL of bloody pericardial fluid was drained. A pericardial drain was placed for continuous drainage, and an additional 150 mL of pericardial fluid was drained over the subsequent 24 hours, bringing the total drainage volume to 1400 mL. Following the procedure, the patient’s hemodynamic status improved significantly, with a resolution of tamponade physiology.

Post-procedure course

**Figure 2:**
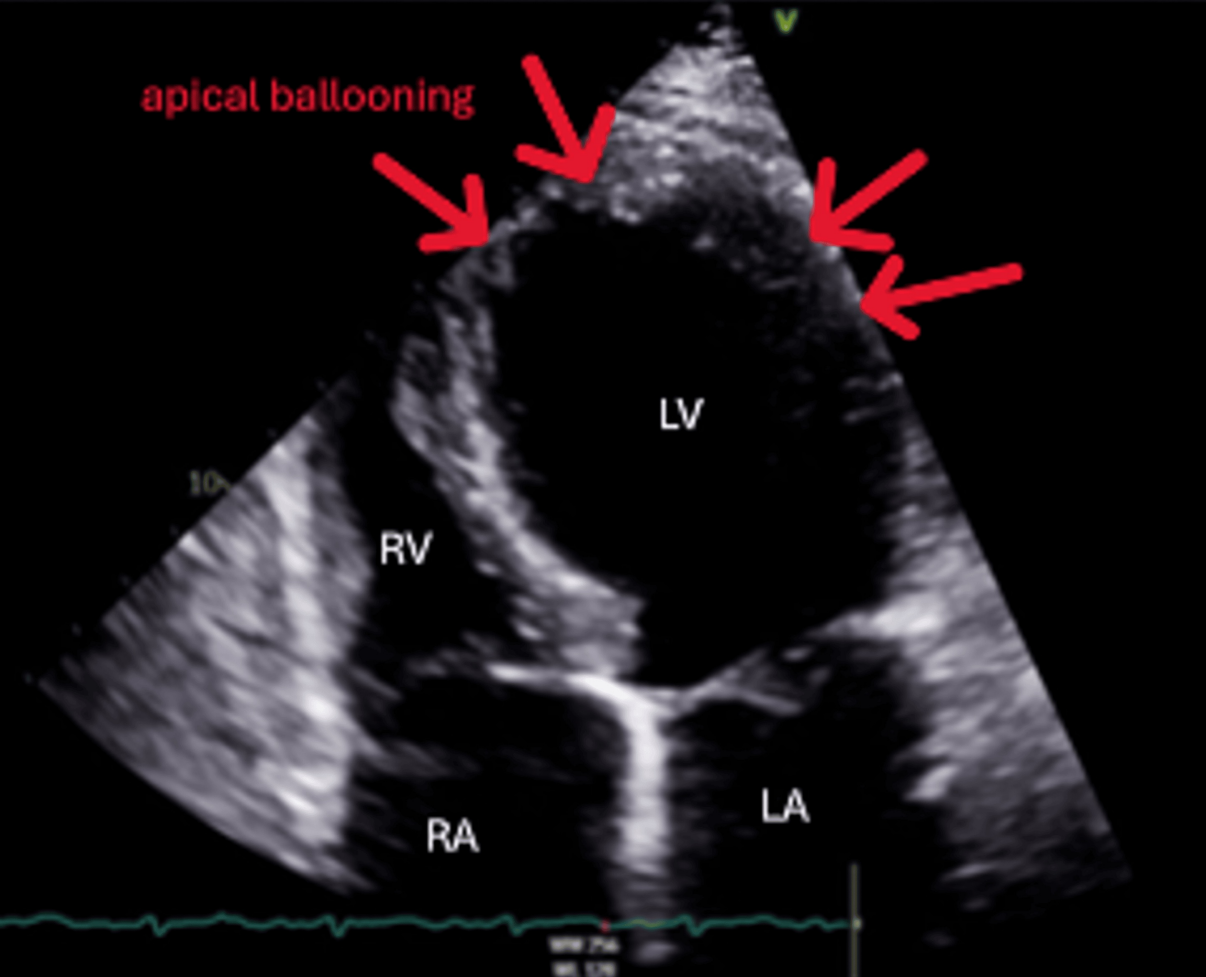
Regional wall motion abnormalities with akinesia of the apex and peri-apical areas (apical ballooning pattern)

Echocardiography was repeated the following day after pericardiocentesis to reassess the pericardial effusion. It revealed a moderately sized pericardial effusion. However, the more significant finding was the incidental discovery of new regional wall motion abnormalities in the left ventricle (LV), presenting as akinesia of the apex and peri-apical areas (apical ballooning pattern). The LV systolic function was severely impaired due to these new changes, resulting in an ejection fraction (EF) of 25-30%. Mild to moderate right ventricular (RV) systolic dysfunction was also noted (Figure 2, Videos 1, 2).

**Video 1:** ECHO showing regional wall motion abnormalities in the LV and an apical ballooning pattern ECHO: echocardiogram; LV: left ventricle

**Video 2:** ECHO: different view ECHO: echocardiogram

**Figure 3:**
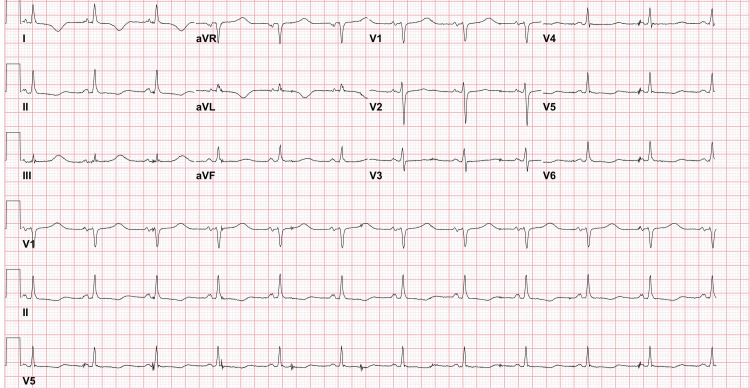
ECG changes post pericardiocentesis Nonspecific ST-T changes, with significant QTc prolongation compared to baseline ECGs ECG: electrocardiogram

Follow-up ECGs revealed nonspecific ST-T changes, with significant QTc prolongation compared to baseline ECGs (Figure 3).

Laboratory tests showed a significant elevation in Troponin-T values, reaching up to 1892 ng/L, as well as an elevation in BNP, which reached up to 6829 pg/ml. Collectively, the new findings from the repeated echocardiogram, along with the ECGs and lab values, were highly suggestive of Takotsubo cardiomyopathy (TC). Accordingly, the patient was started on appropriate heart failure medications, including Bisoprolol 2.5 mg OD and Valsartan 40 mg BID. As part of the plan, a follow-up echocardiogram was scheduled to be done soon thereafter. Over the course of her hospital stay, her symptoms improved significantly. Five days later, a follow-up echocardiogram was performed, and it showed significant recovery of bi-ventricular systolic function, with notable improvement in the previously observed regional wall motion abnormalities. There was only mild pericardial effusion (Video 3).

**Video 3:** ECHO after the resolution of Takotsubo cardiomyopathy ECHO: echocardiogram

Coronary angiography was initially planned as part of the workup. However, given the rapid recovery of LV systolic function and significant improvement of the regional wall motion abnormalities, the diagnosis of TC was established.

Outcome and follow-up

The patient’s pericardial effusion drain was reduced to nil, after which the pigtail was removed, and the patient was discharged in stable condition on the following medications: Bisoprolol 2.5 mg, Sevelamer 2400 mg, and Valsartan 80 mg BID. A follow-up appointment was scheduled, with a repeat echocardiogram to be performed in six weeks.

## Discussion

This case highlights a relatively rare complication of a commonly performed procedure [3]. A complication that’s both critical to detect and easily reversible and resolvable, TC will present as a reversible, stress-induced left ventricular dysfunction that occurs due to physical or emotional stress [1]. Its definitive features include echocardiographic findings of transient regional wall motion abnormalities of LV or RV myocardium such as LV-basal hypercontractility and apical ballooning, a sudden, significant, and otherwise unexplainable drop in ejection fraction in the absence of culprit coronary artery disease; ECG abnormalities, a sudden elevation in BNP and Troponin T, and ending with the full resolution of the dysfunction and full recovery [1]. Pericardiocentesis is a life-saving procedure, which is generally regarded as a safe procedure; however, it is not without its complications. It is routinely used as a treatment option for pericardial effusion and is the definitive management for cardiac tamponade [4]. This case highlights the paradoxical effect of a procedure meant to relieve stress on the heart causing additional stress and inducing cardiac dysfunction. It adds to the limited body of evidence suggesting that after pericardiocentesis, physicians should maintain a higher index of suspicion for the possibility of progression to TC, particularly in higher-risk comorbid populations, such as those with hyperlipidemia, hypertension, smoking, alcohol abuse, anxiety, depression, and stress [5].

Previous literature

Similar cases of TC have been reported post-pericardiocentesis, such as by Zaclli (2024, US) [6], Bhutani (2022, India) [4], and Versaci (2015, Italy) [7]. Other cases have been reported after other types of procedures such as TC post-pericardiectomy (Zhang (2021, China) [8]) and post-cardiac surgery (Chiariello (2016, Italy) [2]). We believe that this warrants further studies to better understand the pathophysiological link between these interventions and the resulting pathology and to guide more specific management in future cases.

## Conclusions

This case highlights Takotsubo cardiomyopathy as a rare but significant complication of pericardiocentesis. It adds to the limited body of research addressing this finding. Given the scarcity of available literature, this complication could be mismanaged, misdiagnosed, or missed completely. Clinicians should maintain a relatively higher index of suspicion for TC in post-procedure patients, particularly those with multiple comorbidities. Timely recognition and appropriate management can result in more favorable outcomes.
